# Detection of filamentous tau inclusions by the fluorescent Congo red derivative FSB [(*trans*,*trans*)-1-fluoro-2,5-bis(3-hydroxycarbonyl-4-hydroxy)styrylbenzene]

**DOI:** 10.1016/j.febslet.2008.02.025

**Published:** 2008-03-19

**Authors:** Ana Velasco, Graham Fraser, Patrice Delobel, Bernardino Ghetti, Isabelle Lavenir, Michel Goedert

**Affiliations:** a Medical Research Council Laboratory of Molecular Biology, Hills Road, Cambridge CB2 0QH, UK; b Department of Pathology and Laboratory Medicine, Indiana University School of Medicine, 635 Barnhill Drive, Indianapolis, IN 46202, USA

**Keywords:** Amyloid, Congo red, Filamentous inclusion, Live imaging, Tauopathies

## Abstract

Filamentous inclusions made of the microtubule-associated protein tau in a hyperphosphorylated state are a defining feature of a large number of human neurodegenerative diseases. Here we show that (*trans*,*trans*)-1-fluoro-2,5-bis(3-hydroxycarbonyl-4-hydroxy)styrylbenzene (FSB), a fluorescent Congo red derivative, labels tau inclusions in tissue sections from a mouse line transgenic for human P301S tau and in cases of familial frontotemporal dementia and sporadic Pick’s disease. Labelling by FSB required the presence of tau filaments. More importantly, tau inclusions in the spinal cord of human P301S tau transgenic mice were labelled following a single intravenous injection of FSB. These findings indicate that FSB can be used to detect filamentous tau in vivo.

## 1 Introduction

The most common neurodegenerative diseases are characterized by the presence of abnormal filamentous protein inclusions in nerve cells of the brain [1]. In Alzheimer’s disease (AD), these inclusions are made of hyperphosphorylated tau protein. Together with the extracellular β-amyloid deposits, they constitute the defining neuropathological characteristics of AD [2]. Tau inclusions, in the absence of extracellular deposits, are characteristic of progressive supranuclear palsy, corticobasal degeneration, Pick’s disease, argyrophilic grain disease and frontotemporal dementia and parkinsonism linked to chromosome 17 (FTDP-17T) [3]. The identification of mutations in *Tau* in FTDP-17T has established that dysfunction of tau protein is central to the neurodegenerative process [4–6].

The realisation that the formation of abnormal protein deposits, such as β-amyloid and tau, precedes clinical symptoms, has led to attempts at developing reliable biomarkers for neurodegenerative diseases. At present, work is most advanced for AD, where functional neuroimaging is showing great promise for detecting pathological deposits, in particular β-amyloid, in asymptomatic patients and in patients in the early stages of the disease [7,8]. A number of positron emission tomography tracer chemotypes is currently under investigation, including [^18^F]-FDDNP, [^11^C]-PIB, [^11^C]-SB13 and [^11^C]-BF227 [9]. Most tracers are Congo red or thioflavin derivatives, in keeping with the amyloid nature of the pathological deposits [10,11]. These ligands, with the exception of [^18^F]-FDDNP, display similar uptake in cerebral cortex of AD patients, consistent with in vitro data showing that they have a high affinity for β-amyloid. [^18^F]-FDDNP also displays increased tracer uptake in the medial temporal lobe, suggestive of a possible interaction with tau deposits [12].

Currently, only little is known about the ability of these and related compounds to detect tau inclusions in brain. Previous work has shown that the Congo red derivative (*trans*,*trans*)-1-bromo-2,5-bis(3-hydroxycarbonyl-4-hydroxy)styrylbenzene (BSB) can detect β-amyloid deposits in transgenic mouse brain in vivo and in *post mortem* mouse and human brain [13]. This compound has also been shown to detect tau inclusions in tissue sections from human brain [14]. However, it failed to detect inclusions in some tauopathies, including the neuronal deposits in a case of FTDP-17T with the P301L mutation in *Tau*. Subsequently, (*trans*,*trans*)-1-fluoro-2,5-bis(3-hydroxycarbonyl-4-hydroxy)styrylbenzene (FSB), a fluoro derivative of BSB, was shown to be a more sensitive probe than BSB for detecting β-amyloid deposits in tissue sections and in vivo [15,16] and for detecting tau inclusions in AD brain [15].

Here we have used a previously described [17] transgenic mouse model of human tauopathy and cases of human tauopathies to investigate the usefulness of FSB for detecting tau inclusions. These mice express one isoform of human four-repeat tau with the P301S mutation that causes FTDP-17T [18], under the control of the murine Thy1 promoter. They develop abundant deposits of filamentous tau throughout the central nervous system (CNS), with the greatest load in the spinal cord. We show labelling of filamentous tau by FSB in *post mortem* tissue from mouse and human CNS and in transgenic mouse spinal cord in vivo.

## 2 Materials and methods

### 2.1 Animals and antibodies

Five month-old mice transgenic for human P301S tau [17], eight month-old mice transgenic for the 695 amino acid isoform of the human amyloid precursor protein with mutations K670M/N671L + V717F (line CRND8) [19] and age-matched C57BL/6J controls were used. We made use of the phosphorylation-dependent anti-tau antibodies AT8 (Innogenetics, Ghent, Belgium) and AT100 (Innogenetics) and of the phosphorylation-independent anti-tau antibodies BR134 [20] and T14 (Zymed, San Francisco, CA). All antibodies were used at 1:500. AT8 recognizes tau protein phosphorylated at S202 and T205 [21], with AT100 requiring phosphorylation of T212, S214 and T217 [22]. BR134 (directed against the C-terminus of tau) recognizes murine and human tau isoforms irrespective of phosphorylation [20], whereas T14 is specific for human tau (recognizing residues 141–149) [23].

### 2.2 Histochemistry and immunohistochemistry

Mice were perfused transcardially with 4% paraformaldehyde in 0.1 M phosphate buffer, pH 7.2. Brains and spinal cords were removed, postfixed overnight and cryoprotected in 30% sucrose in phosphate buffer for 24 h. Sagittal brain sections and transverse sections of the spinal cord (30 μm) were cut on a Leica SM2400 microtome (Leica Microsystems, Bucks, UK) and stored at 4 °C in phosphate buffered saline (PBS) containing 0.1% sodium azide. Sections were permeabilized and blocked for 3 h at room temperature in PBS containing 3% bovine serum albumin and 0.1% Triton X-100. For staining with FSB (Dojindo Laboratories, Kumamoto, Japan), sections were incubated for 30 min in blocking solution. For immunohistochemistry, sections were incubated overnight at 4 °C with the primary antibodies in blocking solution. After washing the sections were incubated for 3 h at room temperature in Cy3 (Stratech Scientific, Newmarket, UK) secondary antibodies in blocking solution. Paraffin-embedded sections (10 μm) of formalin-fixed brain tissue from the frontal cortex of a case of FTDP-17T with the P301L mutation in *Tau* [24] and a case of sporadic Pick’s disease were also used. The sections were deparaffinized and rehydrated. Antigen retrieval was performed by incubating the sections in 10 mM sodium citrate buffer, pH 6.0, for 15 min in a microwave oven. Endogenous peroxidase activity was quenched during a 90 min incubation in 20% methanol/3% hydrogen peroxide. Following permeabilization and blocking in Tris-buffered saline-0.2% Tween containing 5% goat serum, the sections were incubated overnight at 4 °C with the primary antibodies in blocking solution. After washing, the sections were incubated for 2 h at room temperature with the Alexa Fluor 568 secondary antibody (Molecular Probes). Following washing, the signal was revealed with the tyramide signal amplification kit. Sections were mounted using Vectashield (Vector Laboratories, Burlingame, CA) and viewed through a Biorad Radiance 2000 confocal microscope (Biorad, Hertford, UK).

### 2.3 Binding of FSB to synthetic tau filaments

Expression and purification of human P301S tau protein was done as described [25]. Purified recombinant protein (3 mg/ml) was incubated in the presence or absence of heparin (400 μg/ml; Sigma–Aldrich, Suffolk, UK) in 25 μl of 30 mM MOPS, 1 mM 4-(2-aminoethyl)benzenesulphonylfluoride (AEBSF, Calbiochem), pH 7.4, at 37 °C for 72 h, as described [26]. Aliquots were placed onto carbon-coated 400-mesh grids and stained with 1% lithium phosphotungstate. Micrographs were recorded at a nominal magnification of X40,000 on a Philips model EM208S electron microscope. Intrinsic fluorescence was measured using 0.75 μl tau filament assembly mixture and 0.63 μM FSB in 10 mM sodium phosphate, 1 mM EDTA, pH 7.4, supplemented with 10% ethanol. After 1 h, intrinsic fluorescence was measured in a Ultra Evolution 384 plate reader (Tecan, Reading, UK) using a 340–440 nm filter pair.

### 2.4 Detection of tau inclusions in vivo

Human P301S tau mice were injected intravenously with 300 μl of 0.1% FSB (2.4 mM) in PBS containing 2% mouse albumin. After various times, the mice were perfused transcardially with 4% paraformaldehyde in 0.1 M phosphate buffer, pH 7.2. Spinal cords were removed, postfixed overnight and cryoprotected for 24 h. Transverse sections (20 μm) were cut using a Leica CM3050S cryostat (Leica Microsystems) and analysed by confocal microscopy.

## 3 Results

### 3.1 Detection of filamentous tau inclusions by FSB in a mouse model of human tauopathy

**Fig. 1:**
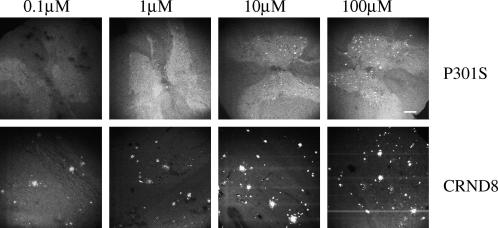
Labelling of tau inclusions and β-amyloid deposits by FSB in transgenic mice. Spinal cord sections from 5 month-old mice transgenic for human P301S tau and brain sections from 8 month-old mice transgenic for human amyloid precursor protein with mutations K670M/N671L + V717F (line CRND8) were incubated with FSB at the indicated concentrations. Confocal microscopy was used to visualize the signal. Scale bar, 150 μm.

**Fig. 2:**
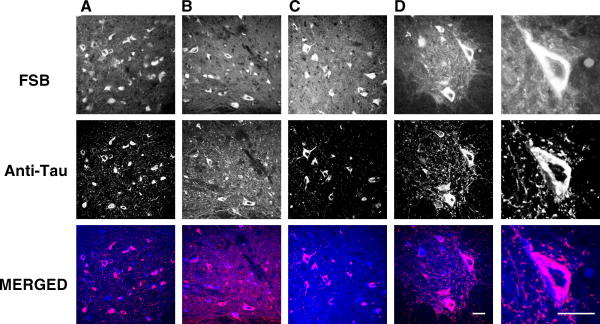
FSB and anti-tau antibodies label the same structures in mice transgenic for human P301S tau. Spinal cord sections from 5 month-old transgenic mice were incubated with anti-tau antibodies BR134 (A), T14 (B), AT100 (C) and AT8 (D). Following immunohistochemistry, the sections were incubated with FSB. Confocal microscopy was used to visualize the signal. Scale bar, 60 μm.

**Fig. 3:**
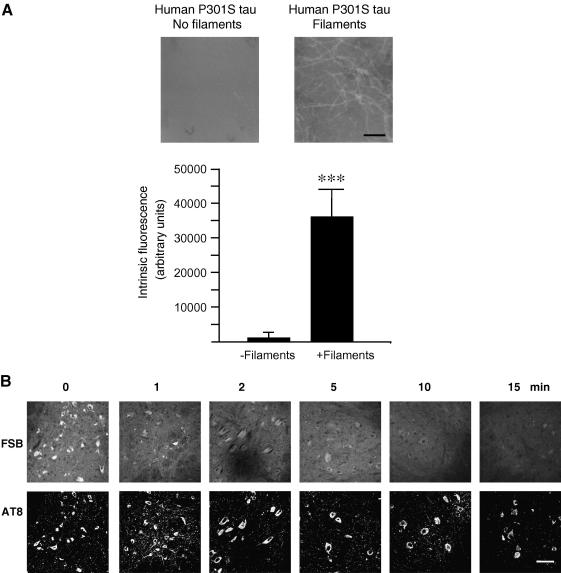
FSB labels filamentous tau: (A) Recombinant human P301S tau protein was incubated in the absence (No filaments) or the presence (filaments) of heparin and labelled with FSB. Intrinsic fluorescence was then measured. Scale bar, 300 nm. (B) Spinal cord sections from 5 month-old human P301S tau mice were incubated with 90% formic acid. Following immunohistochemistry with anti-tau antibody AT8, the sections were incubated with FSB. Scale bar, 60 μm.

Spinal cord sections from 5 month-old mice transgenic for human P301S tau with numerous filamentous tau inclusions were incubated with FSB at concentrations ranging from 0.1 to 100 μM. Strong fluorescence was observed when FSB was used at 10 and 100 μM, with weak labelling at 0.1 and 1 μM (Fig. 1). Previous work has shown that FSB can detect β-amyloid deposits in transgenic mouse brain [16]. We have confirmed these findings using brain sections from mouse line CRND8, where FSB visualized plaques when used at 0.1, 1, 10 and 100 μM. To investigate whether FSB was staining tau inclusions in the spinal cord of transgenic human P301S tau mice, we performed double labelling with anti-tau antibodies and FSB. As shown in Fig. 2, FSB labelled the same cells with tau inclusions as anti-tau antibodies BR134, T14, AT100 and AT8. At higher magnification, FSB and AT8 labelled tau inclusions to the same extent (Fig. 2D). To investigate whether aggregation of tau was required for the binding of FSB, we used assembled and non-assembled recombinant human P301S tau protein (Fig. 3A). The measurement of intrinsic fluorescence showed binding of FSB to filamentous, but not monomeric, tau. This was confirmed using spinal cord sections from human P301S tau mice treated with 90% formic acid. The latter is known to disassemble filamentous inclusions [27]. Pretreatment with formic acid for 1–15 min gradually eliminated the staining of tau inclusions by FSB. By contrast, staining with the phosphorylation-dependent anti-tau antibody AT8 remained unchanged.

### 3.2 Detection of filamentous tau inclusions by FSB in human tauopathies

**Fig. 4:**
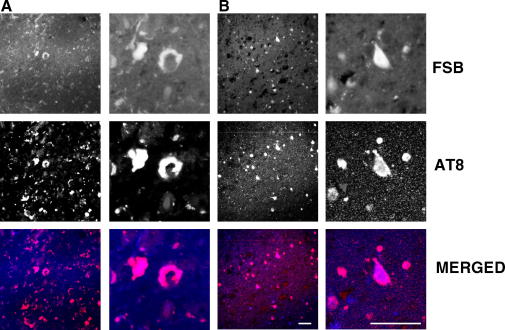
FSB and anti-tau antibody AT8 label the same structures in human tauopathies. (A) Frontal cortex section from a case of FTDP-17T with the P301L mutation in *Tau*. (B) Frontal cortex section from a case of sporadic Pick’s disease. Following immunohistochemistry, the sections were incubated with FSB. Scale bars, 60 μm.

Frontal cortex sections from a case of FTDP-17 T with the P301L mutation in *Tau* and a case of sporadic Pick’s disease were used. Immunohistochemistry for phosphorylated tau was followed by incubation with FSB. As shown in Fig. 4, tau-positive structures were labelled by FSB in both FTDP-17T and Pick’s disease.

### 3.3 Detection of filamentous tau inclusions following the intravenous injection of FSB in transgenic mice

**Fig. 5:**
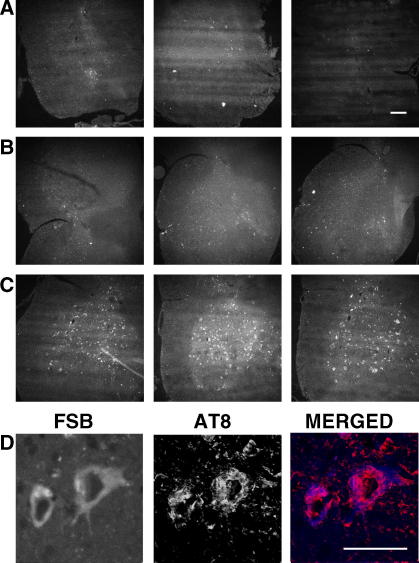
FSB labels filamentous tau inclusions in vivo. Five month-old human P301S tau transgenic mice received a single intravenous injection of 0.1% FSB. One hour (A), 2 h (B) and 4 h (C) after the injection the mice were perfused, the spinal cord sectioned and analysed by confocal microscopy. Scale bar, 150 μm. (D), Motor neurons labelled following the intravenous injection of FSB were also immunoreactive with anti-tau antibody AT8. Scale bar, 60 μm.

To investigate whether FSB could be used to detect tau inclusions *in vivo*, we injected 5 month-old human P301S transgenic mice intravenously with 300 μl of 0.1% FSB (2.4 mM). The mice were killed at various times after the injection, with at least 3 mice per time point. As shown in Fig. 5, numerous nerve cells in spinal cord were labelled 4 h after the injection, with weaker labelling at 2 h. Immunohistochemistry using anti-tau antibody AT8 showed that FSB labelled tau-immunoreactive nerve cells (Fig. 5D).

## 4 Discussion

We have used a mouse line transgenic for human P301S tau protein [17] that exhibits the essential features of human tauopathies to study the usefulness of the Congo red derivative FSB for detecting filamentous tau inclusions. In tissue sections of spinal cord from 5 month-old transgenic mice, FSB readily detected tau inclusions when used at a concentration in excess of 10 μM. Previously, the related compound BSB was used to detect tau inclusions in brain sections from human tauopathies [13,14]. We also detected tau inclusions in brain and spinal cord sections from human P301S tau transgenic mice using BSB, but found it to be less sensitive at detecting inclusions than FSB (data not shown). BSB and FSB also detected amyloid plaques in the brain of transgenic CRND8 mice, in agreement with previous results [13,14,16]. Their ability to detect plaques was greater than that to detect tau inclusions. It remains to be seen whether this is due to a higher intrinsic binding affinity for plaques or whether the extracellular location of plaques and their larger size also play a role.

In tissue sections from transgenic mice, FSB labelled the same structures as phosphorylation-dependent anti-tau antibodies. However, unlike these antibodies, it only recognized tau protein in a filamentous state. In the mouse line transgenic for human P301S tau protein, filamentous inclusions are made predominantly of a single four-repeat human tau isoform [17]. This contrasts with cases of FTDP-17T caused by the P301L mutation in *Tau*, where the three four-repeat tau isoforms make up the inclusions [24] and with sporadic Pick’s disease, where the three tau isoforms with three repeats each make up the filaments [28]. In frontal cortex from a case with the P301L mutation in *Tau* and a case of Pick’s disease, FSB labelled tau inclusions, indicating that it binds to all human brain tau isoforms in a filamentous state. When compared to a previous study reporting only weak labelling of Pick bodies by BSB and no labelling of neuronal inclusions in a case with the P301L mutation in *Tau* [14], the present findings show conclusively that FSB is a more sensitive probe for detecting tau filaments than BSB.

The ability to identify tau inclusions in vivo is essential for experimental studies in animals and for diagnostic purposes in humans [29,30]. We found that FSB can readily label tau inclusions in transgenic mice following a single intravenous injection, demonstrating that it can be used for the live imaging of tau pathology. Like Congo red, FSB has previously been shown to inhibit the heparin-induced filament formation of tau in vitro [31]. It remains to be seen whether the repeated administration of FSB can reduce the formation of filamentous tau inclusions in brain and spinal cord of mice transgenic for human P301S tau.
